# Harnessing Sulforaphane Potential as a Chemosensitizing Agent: A Comprehensive Review

**DOI:** 10.3390/cancers16020244

**Published:** 2024-01-05

**Authors:** Bethsebie Lalduhsaki Sailo, Le Liu, Suravi Chauhan, Sosmitha Girisa, Mangala Hegde, Liping Liang, Mohammed S. Alqahtani, Mohamed Abbas, Gautam Sethi, Ajaikumar B. Kunnumakkara

**Affiliations:** 1 Cancer Biology Laboratory, Department of Biosciences and Bioengineering, Indian Institute of Technology Guwahati, Guwahati 781039, India; bethsailo@gmail.com (B.L.S.); suravi@rnd.iitg.ac.in (S.C.); sosmi176106101@iitg.ac.in (S.G.); mangala.hegde@rnd.iitg.ac.in (M.H.); 2 Department of Gastroenterology, Shenzhen Hospital, Southern Medical University, Shenzhen 518001, China; 1402744723@smu.edu.cn; 3 Guangzhou Key Laboratory of Digestive Diseases, Department of Gastroenterology and Hepatology, Guangzhou Digestive Disease Center, Guangzhou First People’s Hospital, School of Medicine, South China University of Technology, Guangzhou 510180, China; lipingliang13@163.com; 4 Radiological Sciences Department, College of Applied Medical Sciences, King Khalid University, Abha 61421, Saudi Arabia; mosalqhtani@kku.edu.sa; 5 Electrical Engineering Department, College of Engineering, King Khalid University, Abha 61421, Saudi Arabia; mabas@kku.edu.sa; 6 Department of Pharmacology and NUS Centre for Cancer Research, Yong Loo Lin School of Medicine, National University of Singapore, Singapore 117600, Singapore

**Keywords:** phytochemicals, sulforaphane, cancer, chemoresistance, chemosensitization

## Abstract

**Simple Summary:**

Recent oncological research highlights the promising role of naturally derived compounds in cancer prevention and treatment. Sulforaphane (SFN), a phytochemical found in cruciferous vegetables, is a powerful chemosensitizer that increases the sensitivity of cancer cells to chemotherapy and overcomes chemoresistance. When coupled with conventional chemotherapeutic drugs, SFN regulates numerous signaling pathways, proteins and genes which results in synergistic inhibition of cancer progression. The therapeutic potential of SFN is ongoing, with particular emphasis on its chemosensitizing potential against various cancer types.

**Abstract:**

Recent advances in oncological research have highlighted the potential of naturally derived compounds in cancer prevention and treatment. Notably, sulforaphane (SFN), an isothiocyanate derived from cruciferous vegetables including broccoli and cabbage, has exhibited potent chemosensitizing capabilities across diverse cancer types of bone, brain, breast, lung, skin, etc. Chemosensitization refers to the enhancement of cancer cell sensitivity to chemotherapy agents, counteracting the chemoresistance often developed by tumor cells. Mechanistically, SFN orchestrates this sensitization by modulating an array of cellular signaling pathways (e.g., Akt/mTOR, NF-κB, Wnt/β-catenin), and regulating the expression and activity of pivotal genes, proteins, and enzymes (e.g., p53, p21, survivin, Bcl-2, caspases). When combined with conventional chemotherapeutic agents, SFN synergistically inhibits cancer cell proliferation, invasion, migration, and metastasis while potentiating drug-induced apoptosis. This positions SFN as a potential adjunct in cancer therapy to augment the efficacy of standard treatments. Ongoing preclinical and clinical investigations aim to further delineate the therapeutic potential of SFN in oncology. This review illuminates the multifaceted role of this phytochemical, emphasizing its potential to enhance the therapeutic efficacy of anti-cancer agents, suggesting its prospective contributions to cancer chemosensitization and management.

## 1. Introduction

Oncological disorders are highlighted by their marked prevalence, heterogeneity and repercussions on patient well-being and socio-economic burdens [1,2,3]. The initiation of carcinogenesis is characterized by aberrant cellular proliferation, a consequence of genomic perturbations. As these malignancies progress, they manifest capabilities for enhanced self-renewal, proliferation, angiogenesis, metastasis, and notably resistance to therapeutic modalities, often escaping the canonical cellular signaling mechanisms [4,5]. It has always been a major challenge to tackle this disease, demanding for effective preventive measures and the development of better treatment approaches [6,7]. The current therapeutic interventions for cancer comprise conventional approaches such as surgical resection, radiotherapy, and chemotherapy, as well as adjunctive or alternative strategies like hormone therapy, immunotherapy, and combinatorial therapy [6,8]. The prime focus of these modalities is to suppress neoplastic growth by inhibiting or attenuating proliferative efficiencies. Despite the available treatments, they are associated with drawbacks and challenges, notably the adverse side effects and the emergence of chemoresistance in cancer cells [6,8]. These drawbacks limit the efficacy of the available treatment options in the management of cancer [6,8].

**Figure 1:**
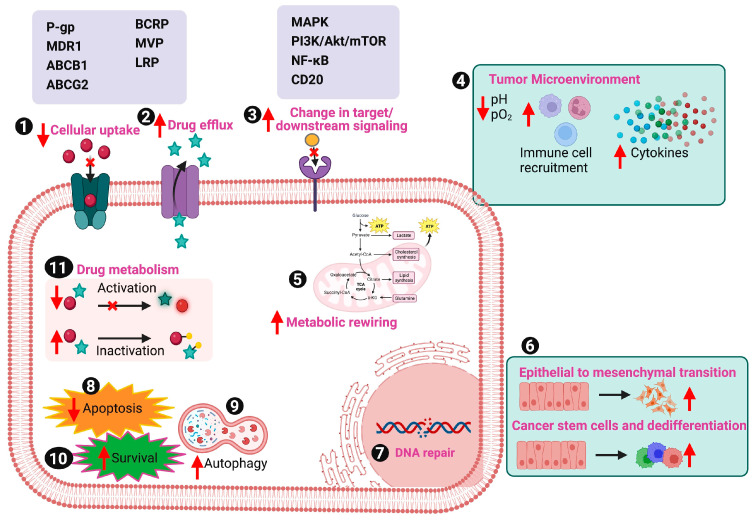
Molecular mechanism of cancer chemoresistance. Chemoresistance manifests through either genetic predisposition or acquired mechanisms. The predominant molecular determinants underlying resistance to cancer therapeutics encompass (1) Altered expression of transport proteins governing drug absorption, resulting in diminished absorption rates and subsequent chemoresistance, (2) Anomalous expression of the ABC family proteins, leading to the efflux of drugs from the cellular milieu, thereby reducing intracellular drug concentrations to levels insufficient for drug sensitivity, (3) Perturbations in targeted signaling pathways, (4) Tumor microenvironmental factors including hypoxia, low pH, elevated cytokine levels, and heterogeneity, (5) Metabolic reprogramming of tumor cells, (6) Induction of epithelial-mesenchymal transition (EMT) properties conferring resistance to chemotherapy and radiotherapy, (7) Prompt repair of DNA damage inflicted by chemotherapy and radiotherapy, closely linked to the acquisition of chemoresistance, (8) Inhibition of cell death processes, (9) Activation of autophagy, (10) Augmented signaling pathways associated with survival, indicating an imbalance between apoptosis and cell growth, modulated by major gene families such as p53 and Bcl, (11) Drug inactivation, wherein detoxification-related proteins deactivate drugs within cells, culminating in the acquisition of chemoresistance.

Chemoresistance, stratified into acquired or intrinsic, is delineated as the adaptive capability of cancerous cells to circumvent the cytotoxic impacts of chemotherapeutic agents, constitutes a principal impediment in ensuring the efficacy of chemotherapy [9]. Such resistance often results in increased tumor progression, invasive and metastatic ability of cancer cells [10,11,12,13]. To devise strategies mitigating chemoresistance, it is pivotal to discern the type and underlying mechanistic pathways fostering it (Figure 1).

Tumor heterogeneity engenders the emergence of aggressive cell populations that exhibit resistance to therapeutic interventions by adapting to chemotherapy and promoting metastasis [13,14]. Additionally, the interactions between anti-cancer drugs and cancer cells induce molecular alterations, reducing drug activation and efficacy thus fostering resistance [15]. Key proteins from the ATP-binding cassette (ABC) transporter family, such as P-glycoprotein (P-gp or multidrug resistance protein 1 (MDR1)/ATP-binding cassette sub-family B member 1 (ABCB1)), breast cancer resistance protein (BCRP or ATP-binding cassette sub-family G member 2 (ABCG2)), and major vault protein (MVP) or lung resistance-related protein (LRP), contribute to chemoresistance by facilitating the efflux of drugs from cancer cells [16,17,18,19,20]. Furthermore, mutations and downregulation in pathways targeted by chemotherapeutic agents like cytarabine (AraC) (used against acute myelogenous leukemia) and enzymatic systems like cytochrome P450 and glutathione-S-transferases affect drug metabolism, leading to chemoresistance [15,21].

Alterations in several genes and signaling pathways, including mitogen-activated protein kinase (MAPK), phosphoinositide 3-kinase (PI3K)/Akt/mammalian target of rapamycin (mTOR), and nuclear factor kappa B (NF-κB), are also associated with chemotherapy resistance [18,22]. For instance, mutations in MAPK pathway components like rat sarcoma (RAS) and v-raf murine sarcoma viral oncogene homolog B1 (BRAF) genes (up to 40% mutations in human tumors such as thyroid cancer and melanoma, lung cancer and pancreatic cancer) contribute to drug resistance [23,24,25]. Additionally, overexpression of P38γ MAPK is linked to chemoresistance, inducing epithelial–mesenchymal transition (EMT) and metastasis in breast cancer [26]. In addition, NF-κB plays a significant role in cancer initiation, progression, and chemoresistance by activating genes that inhibit cell death [27]. Its activation in cancer cells is associated with resistance against drugs like doxorubicin (DOX), cisplatin, and 5-fluorouracil (5-FU) [27]. Genetic mutations or epigenetic changes affecting drug target sites can also lead to resistance [28]. For example, rituximab, targeting a cluster of differentiation (CD20) proteins on B cells, becomes ineffective due to reduced CD20 expression or the activation of survival pathways, causing drug resistance [28]. Moreover, increased activity of nucleotide excision repair (NER) and DNA repair systems counteracts the effects of DNA-damaging chemotherapy agents like cisplatin, resulting in chemoresistance [13,29,30]. Thus, chemoresistance can arise through various pathways, including evasion of cell death, disruptions in signal transduction, and molecular alterations influencing cellular responses to chemotherapy. The intricate interplay of these factors suggests the complexity of overcoming resistance in cancer treatment [13].

To address these challenges, chemosensitization emerges as a crucial approach involving the use of additional agents to enhance the efficacy of primary therapeutic drugs [31]. This strategy aims to render cancer cells more responsive to anti-cancer drugs, overcoming intrinsic or acquired resistance mechanisms by increasing cancer cell susceptibility to chemotherapeutic agents [2,12,32]. Ideally, a chemosensitizer should exhibit minimal toxicity, multi-targeting properties, and the ability to enhance sensitivity across diverse signaling pathways [2]. Chemosensitization approaches encompass combining different agents that target multiple molecular pathways or utilizing agents that modify the tumor microenvironment to sensitize cancer cells, thereby improving treatment outcomes. Ultimately, these approaches aim to enhance the therapeutic impact of chemotherapy and overall treatment responses [33,34].

In this context, phytochemicals have emerged as potential candidates for combination therapy and chemosensitization due to their favorable safety profile, low toxicity, cost effectiveness, and multitargeting property [2,35]. Additionally, phytochemicals can mitigate adverse side effects associated with high-dose administration of other therapeutic agents [2,36,37,38]. Notably, sulforaphane (SFN), a component of cruciferous vegetables, has shown promise as a phytochemical compound capable of inducing chemosensitization in various cancers, including cervical, gastric, lung, ovarian, pancreatic, and prostate cancers [39,40,41]. Moreover, SFN has been reported to enhance the efficacy of other agents and phytochemicals in various combination studies across different cancers [42,43,44,45,46,47]. Furthermore, combinations of SFN with diverse chemotherapy agents and other phytochemicals have been shown to modulate several resistance-related pathways, including MAPK, Akt, NF-κB, signal transducer and activator of transcription 3 (STAT3), and various molecules such as p53, retinoblastoma protein (RB), cyclins, cyclin-dependent kinases (CDKs), matrix metalloproteinases (MMPs), caspases, across different cancer types [48,49,50,51,52].

Therefore, this review highlights the therapeutic potential of sulforaphane as a chemosensitizer and a candidate for combination therapy.

## 2. Sulforaphane

**Figure 2:**
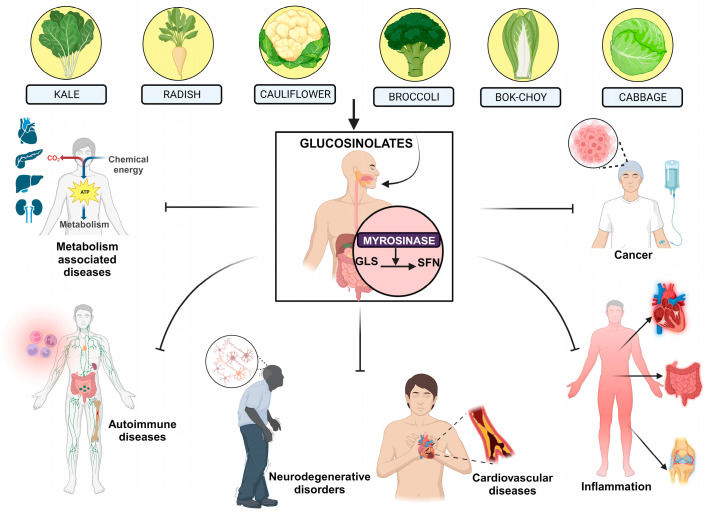
Sources of SFN and its broad spectrum against a wide range of diseases. The different types of cruciferous vegetables are rich in glucosinolates (GLS) like broccoli, kale, cabbage, etc. Under the action of myrosinases present in the gut, GLS becomes converted into sulforaphane SFN, which targets a wide range of diseases like neurogenerative disorders, cancer, autoimmune disorders, cardiovascular diseases, inflammation, metabolic diseases like diabetes, non-alcoholic fatty liver, etc.

SFN (1-isothiocyanate-4-methylsulfinylbutane, an innate isothiocyanate) was first identified by Zhang et al. in 1992 as a primary inducer of phase II enzymes, particularly NAD(P)H:quinone oxidoreductase, in broccoli [53,54]. The precursor of this compound exists in food-bound form as glucoraphanin (GFN), a glucosinolate, and is prevalent in cruciferous vegetables like cauliflower, cabbage, broccoli, kale, radishes, bok choy, etc. [55,56,57] (Figure 2). After the ingestion of these vegetables, both plant-derived myrosinases and endogenous human gut myrosinases initiate the transformation of GFN into SFN [58]. Notably, the sulfoxide group on the GFN side chain can undergo reversible redox reactions, leading to the production of glucoerysolin or glucoerucin. Subsequently, myrosinase mediated hydrolysis results in the formation of SFN analogs, namely erysolin and erucin, respectively (Figure 2) [59].

Studies have reported the chemopreventive and anti-neoplastic properties of SFN in different experimental models [54,56,60]. Pharmacokinetic evaluations in rats demonstrated that, following oral administration of a 50 μM dose, the plasma concentrations of SFN become detectable within one hour and peak at 20 μM by 4 h. In addition, this increase in SFN plasma levels coincides with the upregulation of key genes involved in cellular defense processes and cell cycle modulation, such as metallothionein, glutathione S-transferase A3 (GSTA3), and MAPK in hepatic tissues [56].

### Molecular Target/Anti-Cancer Effect of Sulforaphane

**Figure 3:**
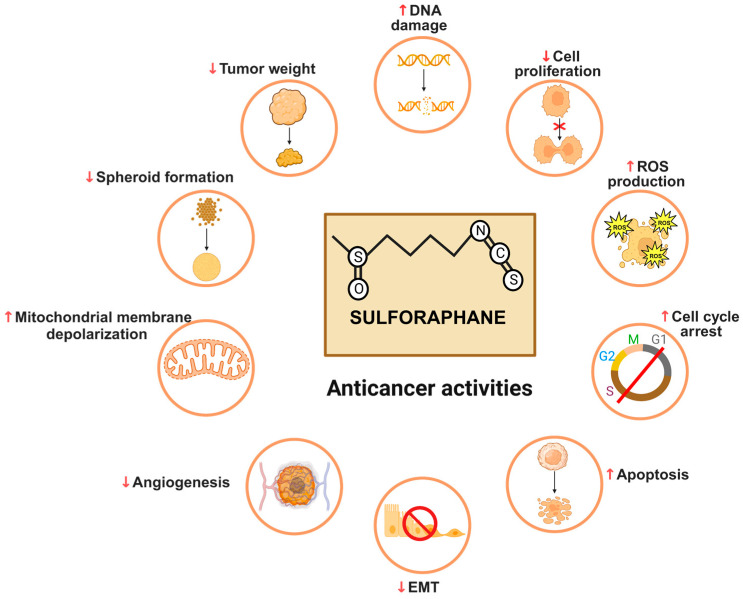
Anti-cancer activities of SFN—Numerous investigations have elucidated the anti-cancer properties of SFN both in vitro and in vivo. SFN elicits anti-cancer effects by inducing DNA damage and ROS production, promoting cell cycle arrest, mitochondrial membrane depolarization, and apoptosis, while concurrently inhibiting proliferation, EMT, angiogenesis, tumor weight, and other related processes.

SFN has attracted substantial attention of various researchers owing to its modulation of multiple molecular pathways associated with oxidative stress, inflammation, cellular proliferation, and apoptosis thus influencing various stages of cancer development [56,60]. Accumulating evidence strongly indicates anti-neoplastic properties of SFN which might be due to its ability to inhibit inflammation, cell cycle progression, angiogenesis and metastasis, and induction of apoptosis and reactive oxygen species (ROS) generation (Figure 3) [61]. SFN functions as an effective histone deacetylase (HDAC) inhibitor, contributing to its cancer-preventive mechanisms which are partially linked to the suppression of phase I detoxification enzymes, including cytochrome P450, family 1, subfamily A, polypeptide 1 (CYP1A1) and CYP2B1/2, and induction of phase II enzymes such as NAD(P)H quinone oxidoreductase 1 (NQO1) and glutathione S-transferase (GST) [62,63].

Additionally, the anti-neoplastic activity of SFN encompasses the elucidation of oxidative stress and the disruption of tubulin polymerization [64]. SFN instigates apoptosis in oncogenic cells via the mitochondrial apoptotic pathway, modulating the expression of proteins like Bcl-2-associated X protein (Bax), Bcl-2 homologous antagonist/killer (Bak), X-linked inhibitor of apoptosis protein (XIAP), and Bcl-2 [65]. Investigations on prostate cancer following SFN exposure found marked downregulation in the expression of inhibitor of apoptosis (IAP) family proteins (including cellular inhibitor of apoptosis protein 1 (cIAP1), cIAP2, and XIAP) with a concomitant upregulation in Apaf-1, resulting in cell death [66].

**Figure 4:**
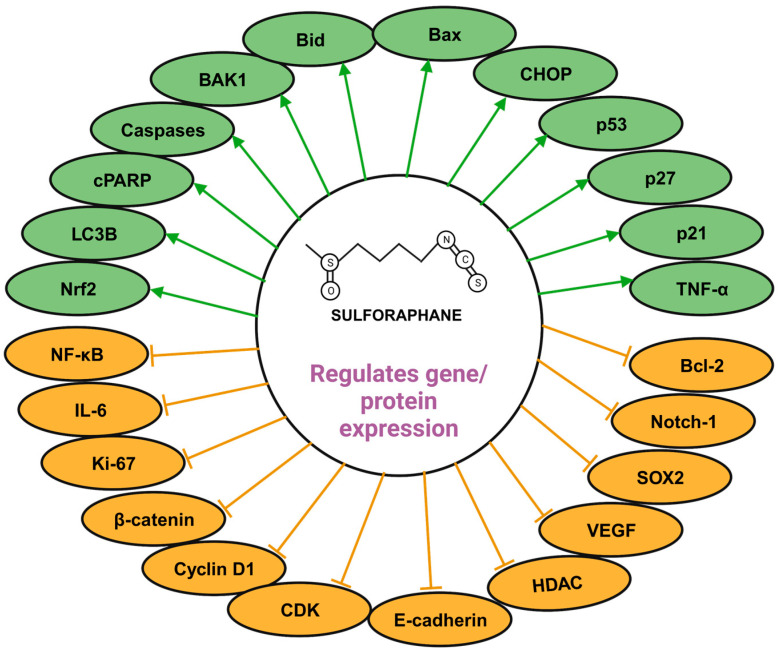
Molecular targets of SFN—SFN exerts its anti-cancer effects by modulating key molecular targets, including caspases, Bid, Bax, p53, Nrf2, p21, NF-κB, cyclin D1, E-cadherin, VEGF, and Bcl-2. This modulation intricately regulates diverse processes in cancer.

From a mechanistic perspective, SFN has been observed to diminish the levels of Ki-67, an established cellular proliferation marker, particularly in prostate and breast malignancies [67]. Several studies highlight SFN’s ability to deter pivotal molecules intrinsic to survival pathways, namely phosphorylated c-Jun N-terminal kinase (p-JNK), phosphorylated extracellular signal-regulated kinases (p-ERK), phosphorylated Akt (p-Akt), and β-catenin, thereby modulating the MAPK, PI3K/Akt, and Wnt signaling pathways [64]. In addition, SFN anti-tumorigenic activities are partly rooted in its anti-inflammatory modulation, notably through the substantial attenuation of NF-κB activity [68]. A salient function of SFN in human physiology is its potentiation of the transcription factor, nuclear factor erythroid 2 (NFE2)-related factor 2 (Nrf2), which exhibits anti-inflammatory effects both directly by activating phase II enzymes and indirectly via the inhibition of the NF-κB signaling cascade [59]. In addition to these molecules, SFN has been reported to modulate various non-coding RNAs such as let-7a, miR-9, miR-23b, miR-145, miR-155, etc., in cancer [69], thus suggesting the therapeutic potential of sulforaphane in the management of cancer by modulating various proteins and genes (Figure 4).

## 3. Chemosensitizing Action of Sulforaphane

**Figure 5:**
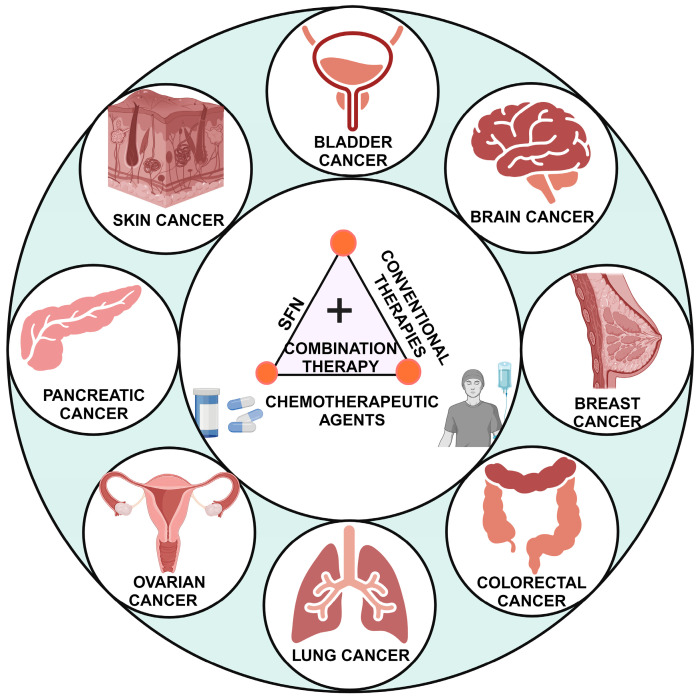
Chemosensitization action of SFN in combination therapy against various cancers—The general overview of combination therapy used against different types of cancers. The treatment uses a combination of SFN, conventional anti-cancer agents, and/or conventional therapies like chemotherapy to achieve chemosensitization.

Novel combinatorial therapies that may improve the efficacy of existing anti-cancer drugs are of urgent requirement to overcome drug resistance in cancer patients. Besides the chemopreventive and potent anti-tumor activity, SFN in combination regimen effectively potentiated the efficiency of other compounds in vitro and in vivo against diverse cancers (Figure 5) (Table 1). Moreover, combinations of SFN with various agents were demonstrated to induce sensitization in different cancer cells to multiple chemotherapeutic agents, as mentioned in Table 1, via targeting diverse pathways and molecules. Thus, the subsequent section explores the combination and chemosensitization potential of SFN against various cancer types.

### 3.1. Bladder Cancer

Bladder cancer arises from the bladder lining, and a substantial number of patients with this carcinoma develop resistance to existing chemotherapeutic approaches which necessitates the development of innovative and more efficacious therapeutic modalities [43,128,129].

Several studies have elucidated the anti-cancer potential of SFN as combination agent in bladder cancer. For example, a combination of carbonic anhydrase inhibitor, acetazolamide (AZ), and SFN is suggested to potentially suppress cell proliferation and enhanced apoptosis in HTB-9 and RT112(H) bladder cancer cell lines by suppressing Akt signaling proteins such as p-Akt, p-mTOR and p-S6 [43]. In the same study, the combination was shown to diminish in vivo tumor growth, accompanied by the downregulation of E-cadherin, N-cadherin, and vimentin [43]. In addition, the long-term administration of the mTOR inhibitor, everolimus, was shown to induce resistance in RT112, UMUC3, and TCCSUP bladder cancer cell lines. However, the combined treatment of everolimus with SFN inhibits drug resistance and significantly decreases cell proliferation via the downregulation of proteins such as p-Akt, p27, p-CDK1, CDK2, cyclin A, and cyclin B, therefore suggesting SFN to be a potential chemosensitizer against drug resistant bladder cancer [71].

TNF-related apoptosis-inducing ligand (TRAIL) resistance is also a common factor in bladder cancer suppressing the process of apoptosis [70]. Interestingly, sulforaphane treatment overcomes this resistance in bladder cancer cells by elevating ROS production, cleaved PARP, cleaved Bid and death receptor 5 (DR5), thereby leading to mitochondrial membrane potential (ΔΨm) loss and induction of apoptosis [70]. Consequently, this study suggests the potential therapeutic efficacy of SFN in conjunction with TRAIL as an adjunctive chemotherapeutic regimen for patients with TRAIL-resistant bladder carcinoma [70]. Hence, the aforementioned studies suggest the potential of SFN as a chemosensitizer and adjunctive, offering a promising therapeutic avenue for the management of bladder cancer.

### 3.2. Brain Cancer

Glioma, a predominant category of malignant cerebral neoplasms, often exhibits resistance to standard therapeutic modalities, including radiotherapy and chemotherapy [62,130]. Hence, strategies are required to overcome these challenges. Studies have shown the potential of SFN in diverse combination studies, functioning both as a chemosensitizer and a combination agent. In line with this, SFN was demonstrated to function as a chemosensitizing agent in temozolomide (TMZ)-resistant glioblastoma cells by enhancing TMZ-induced apoptosis through the modulation of caspase-3, Bcl-2 and Bax expressions as well as suppression of miR-21 levels via Wnt/β-catenin signaling [44]. Similarly, SFN in combination with TMZ was also shown to suppress in vivo tumor growth by regulating Bcl-2 and Bax [44]. Subsequent investigations revealed that SFN chemosensitized U87-R and U373-R glioblastoma cell lines to TMZ by mechanistically suppressing NF-κB activity and O6-methylguanine-DNA methyltransferase (MGMT), and by modulated expressions of caspases, resulting in decreased cell proliferation [64]. Additionally, this effect of the combination was observed in a xenograft model, resulting in decreased tumor volume [64].

Additionally, studies have shown the remarkable efficacy of SFN as combination agent with other drugs and molecules. For example, concomitant treatment of SFN and another polyphenolic compound, resveratrol, on U251 glioma cells significantly attenuated the expression of p-Akt, cyclin D1 and PCNA, while inducing Bax, cytochrome C (Cyt C) and cleaved caspase-3, leading to apoptosis [62]. Further, co-treatment of SFN and a peptide nucleic acid, R8-PNAa15b, in U251 cells induced apoptosis and suppressed cell proliferation by downregulating miR-15b-5p expression [72]. Moreover, SFN in combination with autophagic inhibitor 3-methyladenine (3-MA) was found to suppress cell viability and induce apoptosis by reducing of Bcl-2 and activating Bax and caspases in neuroblastoma [107]. Collectively, these findings indicate the promising potential of SFN for chemosensitization as well as combination therapies that could enhance the management of glioblastoma.

### 3.3. Breast Cancer

Breast carcinoma, with an estimated 2.3 million new cases in 2020, represents the most prevalent malignancy among women and significantly contributes to high global cancer-related mortality [82,131,132,133]. Despite the relative success in the treatment of early-stage breast cancer, it contributed to one in every six cancer-related fatalities among women in 2020 [82]. Therapeutic strategies for breast cancer are determined based on its biological subtype and disease stage, encompassing surgical interventions, radiation therapy, chemotherapy and targeted therapy. Specifically, for the triple-negative breast cancer (TNBC) subtype, which lacks specific receptors, including estrogen receptor (ER)-, progesterone receptor (PR)-, and human epidermal growth factor receptor-2 (HER2)-), chemotherapy is particularly suggested due to its aggressive nature and lack of targeted therapeutic avenues [82,134].

Epigenetic aberrations, including DNA methylation-mediated silencing of tumor suppressor genes (TSGs), have been implicated in the pathogenesis of sporadic breast cancer [73]. The extended use of chemotherapeutic agents such as cisplatin, lapatinib, 5-FU, paclitaxel, etc., leads to resistance, and the efficacy of natural compounds like SFN has been investigated in overcoming this resistance. Approximately 25% of breast cancers overexpress HER2, and the agents targeting HER2 like lapatinib are often compromised by inherent or acquired chemoresistance [74]. Interestingly, treatment of HER2-overexpressing breast cancer cell lines (SKBR-3 and BT-474) with the combination of lapatinib and SFN synergistically induced chemosensitization to lapatinib and decreased cell viability through the suppression of p-HER2, p-Akt, and p-S6 [74]. Similarly, the co-treatment of SFN and 4-hydroxytamoxifen led to chemosensitization and reduced cell viability by inhibiting the expression of Bcl-2 and survivin while increasing Bax, adipose differentiation-related protein (ADRP) and cleaved poly (ADP-ribose) polymerase (PARP) in ER-positive breast cancer cell lines [76]. In addition, SFN treatment enhanced paclitaxel-induced apoptosis and reduced cell viability in breast cancer cells by inhibiting the NF-κB pathway and inducing Cyt C and caspases [50]. Moreover, SFN was found to sensitize triple negative breast cancer cells to taxanes such as paclitaxel and docetaxel (DTX) by inhibiting cell viability and diminishing the expression of cyclin D1, interleukin (IL)-6 and -8, and stem cell markers [42]. In the same study, the combination of SFN and taxanes led to reduced tumor volume and secondary tumor formation in animal models [42]. Further, SFN was found to improve sensitivity to DOX in breast cancer cell lines by inhibiting cell proliferation through the activation of Nrf2, HO-1, caspase, and anti-oxidant enzymes while repressing cyclooxygenase-2 (Cox-2), prostaglandin E2 (PGE2) and HDAC expression [78,79,81,85]. Furthermore, the combination of cisplatin and SFN suppressed metatstasis and cisplatin resistance by downregulating SIRT-initiated EMT by reducing MMPs, N-cadherin, vimentin, Snail, and Slug and upregulating E-cadherin, claudin-1 and zonula occludens-1 (ZO-1) in MDA-MB-231 and MDA-MB-468 cells [86]. In addition, the combination of SFN and DOX encapsulated in an aggregation-induced emission (AIE) with D-alpha tocopheryl polyethylene glycol succinate (TPGS), referred to as (AT/Dox/SFN), reversed multidrug resistance (MDR) by inducing cellular uptake of the drug and lead to the inhibition of cell viability associated with decreased Bcl-2 and increased H2A histone family member X (γ-H2AX), Cyt-c and PARP cleavage [87]. AT/Dox/SFN was also shown to reduce tumor volume, increase drug accumulation, activated caspase-3 and downregulated Ki-67 in animal models [87].

Breast cancer stem cells (BCSCs), identified by the ESA+CD44+CD24- phenotype and known for exhibiting resistance to DTX, can be targeted using nanoparticle formulations to enhance anti-cancer efficacy [75]. In line with this, the in vitro treatment consisting of DTX and SFN-loaded poly (D, L-lactide-co-glycolide)/hyaluronic acid (PLGA-b-HA)-based nanoparticles (DTX-SFN-PLGA-b-HA) effectively increased cytotoxicity in both BCSCs and differentiated breast cancer cells (DBCCs) by downregulating β-catenin [75]. Similar outcomes were observed in animal models, with significant suppression of tumor growth and self-renewal properties of breast cancer stem cells (BCSCs), suggesting therapeutic efficiency of DTX-SFN-PLGA-b-HA for breast cancer [75]. The prolonged application of cisplatin might cause resistance and off-target toxicities, and to address these problems, Xu et al. developed nanoparticle formulations comprising cisplatin and SFN (SFN-CDDP-NPs). Remarkably, this combined formulation was shown to increase specificity towards cancer cells, promoting apoptosis via reduction in Bcl-2 and upregulation of p53 while enhancing PARP cleavage and γ-H2AX activation [83]. The liposomal formulation consisting of DOX and SFN inhibited cell viability and induced accumulation of DOX in the nucleus of breast cancer cells [82]. In the same study, this liposomal formulation was found to reduce tumor volume in animal models, which might be due to suppression of mitosis [82].

Studies have also suggested the efficacy of SFN in combination treatment with other agents and phytochemicals. For example, the combination of SFN and 2′-deoxyadenosine analog, clofarabine (ClF), induce hypomethylation of TSG, notably phosphatase and tensin homolog (PTEN) and retinoic acid receptor-beta 2 (RARβ2) promoters, resulting in increased cell growth arrest and apoptosis [73]. Another study revealed that SFN-ClF combination suppressed proliferation of breast cancer cells by reactivating TSG and cyclin-dependent kinase inhibitor 2A (CDKN2A) [77]. Moreover, the combination of SFN with other phytochemicals such as genistein (GEN) and withaferin A was found to inhibit cell viability and induce cell cycle arrest through the modulation of various proteins like p53, p-RB, p21, cyclin, CDK, and HDAC2 and HDAC3 in breast cancer cells [46,52]. In addition, the co-administration of SFN and GEN resulted in inhibition of tumor growth and tumor incidence in a mouse model [46]. Similarly, the combination of SFN, GEN and sodium butyrate (SFN-GEN-NaB) was found to prevent the development of breast cancer mainly by regulating epigenetic modifications through the suppression of DNA methyltransferase (DNMT), HDAC, histone H3 methylations and inducing the activity of histone acetyltransferases [80]. Further, a nanoformulation of SFN and metformin reduced the survival efficiency of MCF-7, 10, and BT-474 cell lines by modulating the expression of Bcl-2, Bax, Src, Wnt1, β-catenin and CD44 [84]. Overall, these findings support the promising potential of SFN as a combination and a chemosensitizing agent in the treatment and management of breast cancer.

### 3.4. Colorectal Cancer

Colorectal carcinoma (CRC) ranks as the third most common in global cancer incidence and second in mortality. It is highly aggressive and frequently diagnosed among young individuals [135]. Various studies have highlighted the potential of SFN to counteract resistance and enhance the effectiveness of various therapeutic modalities in CRC management. For example, co-treatment of SFN with 5-FU, oxaliplatin and folinic acid (FOLFOX) sensitizes CX-1 cells to this therapy by inducing apoptosis and inhibiting cell viability and spheroid formation via the repression of aldehyde dehydrogenase isoform 1 (ALDH1) levels [97]. In another noteworthy investigation, SFN combined with CB-5083 countered resistance and reduced cell proliferation by inactivating NF-κB in HCT116 CRC cells resistant to CB-5083 [96]. Additionally, administration of SFN with multiple bioactive compounds, including lycopene, quercetin, curcumin, termed as MIX, resulted in enhanced anti-proliferative effects on CRC cells with an additional attribute of preserving normal cellular integrity [95]. MIX, when used in combination with 5-FU or cisplatin, was found to induce sensitivity in CRC cells resulting in reduced cell proliferation [95]. Extending these studies, other combination studies also exhibited remarkable efficacy of SFN employed as co-treatment with other agents. For instance, co-treatment with SFN and the flavonoid apigenin in Caco-2 CRC cell line synergistically amplified the induction of phase II detoxification enzymes, notably glutathione and UDP-glucuronosyltransferases A1 (UGTA1), via the modulation of ERK and NF-κB translocation, thus signifying its chemopreventive potential [92]. Additionally, the combined application of SFN and 3,3′-diindolylmethane (DIM) resulted in a notable synergy in arresting CRC cells at G2/M phase cell cycle arrest [54]. Moreover, combined treatment of SFN and polyphenol (-) epigallocatechin-3-gallate (EGCG) culminated the synergistic activation of the transcription factor, activator protein-1 (AP-1), and reduction in cyclin-D1 expression, thereby reducing cell viability of HT-29 CRC cells [93]. In another study, SFN augmented the apoptotic potential of oxaliplatin (OXP) in Caco-2 CRC cells by inducing cleaved PARP and cleaved caspase-3, and -8 levels [57]. Further, SFN in conjunction with peptide-nucleic acids (PNAs), was found to target miR-15b-5p, which lead to reduced cell growth and induction of apoptotic pathways in HT-29 CRC cells [94]. An innovative therapeutic strategy encompassing SFN combined with *Lactobacillus*-treated peripheral blood mononuclear cells was demonstrated to induce apoptosis in CRC cells via the TNF-α signaling axis [47]. Moreover, it was shown that the combination of salinomycin and SFN exerted a strong synergistic activity against CRC by affecting molecular pathways like Akt signaling and triggering apoptotic responses in experimental studies [49]. Further, SFN in combination with vitamin D was found to induce anti-inflammatory activity and suppress tumorigenesis by regulating epigenetic alterations of genes pivotal for CRC pathogenesis [98]. Collectively, these findings demonstrate the prospect of SFN as an adjunct in CRC therapeutic strategies, inducing sensitivity and augmenting the effectiveness of standard and innovative treatment modalities.

### 3.5. Lung Cancer

Lung carcinoma represents one of the predominant cancers diagnosed globally and is the foremost cause of cancer-related mortality among men, with total statistics of 2.2 million new diagnoses and 1.79 million annual fatalities being reported in 2020 [132,136,137]. A significant clinical challenge in lung cancer therapy is the acquired resistance to chemotherapeutic drugs such as cisplatin and gefitinib [104,105]. Interestingly, the therapeutic potential of SFN has been elucidated in various experimental settings as a combinatorial and chemosensitizing regime for lung cancer treatment. In line with this, SFN was found to enhance the therapeutic outcomes of cisplatin in non-small cell lung cancer (NSCLC) models, both in vitro and in vivo, by sensitizing the cells to cisplatin treatment by downregulating the expression of cellular Myc (c-Myc) [104]. Another pivotal study demonstrated that SFN attenuates gefitinib resistance in lung cancer cells by suppressing the expression of sonic hedgehog, smoothened, zinc finger protein GLI1, and stem cell markers CD133 and CD44, which resulted in inhibition of proliferation of gefitinib-resistant lung cancer cells [105]. In addition, Meng et al. evaluated that T790M mutation in PC-9/AB11 lung adenocarcinoma cells was involved in phenotypic transformation, induction of EMT and resistance to gefitinib treatment. However, SFN treatment restored gefitinib sensitivity in these mutated PC-9/AB11 cells by inducing the expression of epithelial markers while concomitantly downregulating mesenchymal proteins and suppressing PI3K/Akt and epidermal growth factor receptor (EGFR)-ERK signaling cascades [45]. Moreover, the combined action of SFN with AZ was shown to overcome resistance and inhibit tumor growth and proliferation, with significant downregulation of stem cell-related genes in bronchial carcinoma [88]. Furthermore, in a combination study, SFN with another isothiocyanate, allyl isothiocyanate (AITC), synergistically enhanced G2/M phase arrest and apoptosis while inhibiting cell migration and metastasis in A549 lung cancer cells, as evidenced by the reduction in survivin, cyclin-B1 and MMP-9 and upregulation of p53, p21, caspase-3 and PARP cleavage [51]. Therefore, SFN demonstrated notable efficacy in overcoming resistance to chemotherapy drugs and serves as a synergistic agent, offering potential benefits in the management of lung cancer.

### 3.6. Ovarian Cancer

Ovarian carcinoma ranks as the third most prevalent gynaecological malignancy and is characterized by the most severe prognosis, having the highest mortality rate among gynaecologic cancers [138,139]. Although its incidence is lower than that of breast carcinoma, its lethality is approximately three times greater. Projections indicate a marked increase in its mortality, expected to be around 307,000 patients by 2040, primarily attributed to late-stage detections resulting from its often-asymptomatic progression [138,139]. Moreover, due to the rising resistance to conventional therapies, there is a significant need for enhancing the effectiveness of primary treatment methods used against this cancer [110]. Intriguingly, it was demonstrated that SFN induced sensitivity to cisplatin in A2780 and SKOV3 human ovarian carcinoma cell lines through the activation of the Nrf2 signaling pathway [110]. In addition, Gong et al. revealed that SFN restores sensitivity to cisplatin in resistant A2780/CP70 and IGROV1-R10 ovarian carcinoma cell lines and xenograft model [112]. This SFN-mediated reversal of resistance was due to upregulation of the tumor-suppressing miR-30a-3p, which subsequently targeted excision repair crosscomplementing1 (ERCC1) and ATPase copper transporting alpha (ATP7A), thereby amplifying drug uptake [112]. Another noteworthy study showed that SFN aids in overcoming cisplatin resistance mediated by c-Myb in ES2 and OVCAR3 cell lines, suggesting the efficacy of SFN against ovarian cancer [113]. Collectively, these observations indicate the potential of SFN as an adjunct in mitigating cisplatin resistance in ovarian carcinoma [112,113]. Additionally, the co-administration of EGCG and SFN reduced paclitaxel resistance in ovarian cancer cells by promoting apoptosis through the cleavage of PARP and downregulation of hTERT and Bcl-2, thus enhancing therapeutic efficacy [109]. Moreover, combinatorial therapy comprising cisplatin, EGCG, and SFN exhibited sensitivity and enhanced cisplatin-mediated apoptosis and induced G2/M phase cell cycle arrest through the upregulation of p21 [108]. Further, a combination study of SFN with cisplatin plays a synergistic role in A2780 cells by downregulating Bcl-2, cyclin-D1, and c-Myc, accompanied by the elevated expression of tumor suppressor p53 and activation of caspase-3, collectively leading to the inhibition of cell proliferation and the induction of apoptosis [111]. Therefore, SFN plays a crucial role in augmenting the efficacy of chemotherapeutic agents such as cisplatin and demonstrated enhanced anti-cancer activity when combined with other agents.

### 3.7. Pancreatic Cancer

Pancreatic carcinoma, despite advancements in medical diagnostics and improved survival outcomes for numerous cancers, persists as one of the most lethal gastrointestinal malignancies. The primary challenge lies in its late diagnosis, which significantly diminishes survival rates [140]. Current research emphasizes the potential therapeutic role of natural products in pancreatic cancer treatment. Existing literature revealed the presence of cancer stem cells (CSCs) in human pancreatic cancer, which are postulated to contribute to metastatic processes and the development of chemoresistance [114]. Numerous studies have demonstrated the therapeutic efficacy of SFN in chemosensitization and combination treatment against pancreatic cancer. With regard to this, a combined regimen of SFN and sorafenib induce chemosensitization and synergistically eradicates pancreatic CSCs in vitro. This effect is mediated through the downregulation of NF-κB activity, inducing apoptosis without substantial toxicities. In addition, this therapeutic combination also notably diminished tumor growth in pancreatic CSC xenograft models, largely via the suppression of EMT-associated proteins, such as Zeb-1, Twist2, and vimentin [114]. Moreover, SFN in combination with heat shock protein 90 (Hsp90) inhibitor, 7-allylamino 17-demethoxygeldanamycin (17-AAG), potentially sensitized Mia Paca-2 pancreatic cancer cells by reducing their proliferation through the suppression of Hsp90, Raf-1, and Akt while inducing caspase-3 activity [115]. In the same study, it was shown that this combined treatment subsequently reduced tumor growth in vivo [115]. Moreover, studies have demonstrated the efficacy of SFN as adjunctive used with other agents for combination therapy. For example, solid lipid nanoparticles (SLN) encapsulating both curcumin and aspirin, in conjunction with free SFN, synergistically induced apoptosis and decreased cell proliferation in pancreatic cancer cells [116]. Similarly, in another study, a chitosan-mediated delivery system combining aspirin, curcumin, and free sulforaphane (ACS) demonstrated notable tumor suppression in transgenic mouse model [120]. Further in vitro studies have ascertained enhanced apoptosis in pancreatic cancer cells treated with a trifecta of SFN, aspirin, and curcumin (ASN), attributable to upregulated expression of cleaved caspase-3, p-ERK1/2, p-c-Jun, p-p38 MAPK, and p-p53, and suppression of p-Akt and NF-κB [48].

Notably, the co-administration of green tea catechins (GTCs) and SFN yielded significant anti-neoplastic effects by reducing cell viability and migration while elevating apoptosis via inducing miR-let7-a expression and inhibition of K-ras and MMPs in pancreatic cancer cells [118]. Subsequently, a low-dose combination of SFN with anti-histamine, loratadine (LOR), within a self-micro emulsifying drug delivery system (SMEDDS) demonstrated enhanced anti-cancer efficacy, reducing IC50 values in comparison to standalone LOR treatments in pancreatic cancer cell lines. This formulation further exhibited the potential to hinder cell viability and colony formation in Panc-1 and Mia Paca-2 cell lines [117,119]. Thus, these studies highlight the remarkable potential of SFN in sensitizing pancreatic cancer cells to chemotherapy drugs and other combinatorial therapeutics for the management of this cancer.

### 3.8. Prostate Cancer

Prostate carcinoma is one of the prominent malignancies in males, playing a key role in high mortality rates among men globally. Both initial and advanced stages of the disease contribute to therapeutic challenges and subsequent mortality [141]. SFN has been shown to substantially enhance the anti-neoplastic impact of other agents when used in combination against prostate cancer. In line with this, SFN in combination with paclitaxel induced apoptosis in PC-3 cells by modulating the expression of caspase-14 [122]. In addition, a combination approach of SFN with TRAIL synergistically eliminate prostate cancer stem-like cells in vitro by attenuating TRAIL-induced NF-κB binding and downregulating markers like C-X-C chemokine receptor type 4 (CXCR-4), jagged1, sex determining region Y-box 2, Notch 1, and Nanog [121]. Additionally, the self-renewal potential of these cells was compromised due to inhibited ALDH1 activity [121]. In the same study, the combination of SFN with TRAIL was found to reduce tumor growth without pronounced adverse effects, underscoring SFN’s potential to enhance the cancer-specific targeting activity of TRAIL in CSCs [121]. Thus, the therapeutic efficacy of SFN could potentially serve as a combination agent, enhancing the activity of another drug and offering potential benefit in the management of prostate cancer.

### 3.9. Skin Cancer

Malignant melanoma, a highly prevalent cancer globally, is characterized by its aggressiveness, high metastatic potential, and resistance to various cytotoxic agents [18,124]. Epidemiological investigations have suggested a potential reduction in melanoma incidence by dietary phytochemicals [124]. Various experimental evidences have suggested the potential efficacy of SFN as chemosensitizer and synergistic agent in combination studies against skin cancer. For example, the combination of SFN and cisplatin was found to induce sensitivity in epidermal squamous cell carcinoma by reducing spheroid formation and cancer stem cells [99]. In the same study, the combination exhibited inhibition in tumor volume correlated to diminished cell viability and spheroid formation, along with activation of caspases [99]. Additionally, the co-administration of SFN and quercetin markedly reduced cell proliferation, invasion, and metastasis in melanoma cells. It also led to a decrease tumor volume in a mouse model, which correlated with the downregulation of MMP-9 [124]. Moreover, the combination of SFN and the epigenetic agent 5-Aza-2′-deoxycytidine (DAC) was found to reduce cell proliferation in melanoma cells by modulating the levels of CCL5, IL33, angiopoietin-2, CD105; VEGF and CCN4 [125]. In another noteworthy study, the amalgamation of SFN with nano-curcumin within a nanogel formulation was observed to elicit cytotoxicity, diminishing the viability of B16-F10 melanoma cells [126]. Furthermore, Fernblock^®^ XP (FB), a patented extract derived from the tropical fern *Polypodium leucotomos*, in combination with SFN, has been shown to exert anti-neoplastic effects on WM115 and WM266-4 melanoma cells. Mechanistically, this combined approach reduced the expression of vascular endothelial growth factor (VEGF), MMP-1, -2, -3, and -9 while also obstructing inflammasome activation and IL-1β production in melanoma cells [127]. Thus, SFN serves as a potential anti-cancer agent that can synergistically enhance the activity of other therapeutic agents employed against skin cancer.

### 3.10. Other Cancers

SFN has also demonstrated therapeutic potential across other malignancies. In particular, a combined regimen of SFN and 5-fluorouracil (5-FU) has been reported to synergistically inhibit the proliferation of 5-FU-resistant salivary gland adenoid cystic carcinoma ACC-M and ACC-2 cells by targeting NF-κB activity [60]. In addition, in HuCCT-1 and TFK-1 cholangiocarcinoma cells, SFN counteracted cisplatin resistance and enhanced apoptosis through the activation of caspase-3 and PARP and concurrent downregulation of Bcl-2 and XIAP [89]. The gemcitabine (GEM)-initiated EMT in intrahepatic cholangiocarcinoma is one of the common causes for resistance. Interestingly, SFN treatment with GEM was shown to inhibit EMT by suppressing cadherins, vimentin, VEGF-A and MMPs in cholangiocarcinoma cells [90]. Similar inhibition of EMT markers was observed in animal models along with the suppression of proliferative marker Ki-67 and tumor volume [90]. These findings elucidate the potential role of SFN in enhancing the effect of chemotherapeutic agents against cholangiocarcinoma.

The co-treatment of SFN and eugenol in HeLa cells demonstrated a dose-dependent therapeutic response, resulting in decreased cell viability attributed to the suppression of Bcl-2, Cox-2, and IL-β, along with increased expression of caspase-3 [91]. Similarly, in the same study, the combined treatment of SFN with eugenol and gemcitabine showed reduced cell viability and an elevated expression in caspase-3 in cervical cancer cells [91].

Moreover, in case of head and neck squamous cell carcinoma (HNSCC), the existing treatment modalities are associated with the limitations, therefore, strategies such as chemosensitization are currently being employed [142]. In line with this, Elkashty and team reported the chemosensitizing properties of SFN in head and neck squamous cell carcinoma by demonstrating its synergistic action with cisplatin and 5-FU, leading to caspase-dependent apoptosis in cancer stem cells without affecting their normal counterparts [101].

Furthermore, SFN was found to counteract resistance in hepatoma cells by inducing TRAIL-mediated apoptosis via ROS-induced amplification of DR5 activity. In addition, SFN was observed to sensitize hepatoma cells overexpressing either B-cell lymphoma-extra-large (Bcl-xL) or Bcl-2 to TRAIL-mediated apoptosis [103].

Multimodal approaches like adjuvant and combination therapy are currently in demand to combat chemoresistance and treat gastric cancer [143]. In line with this, SFN in combination with lapatinib, a small-molecule tyrosine kinase inhibitor, and SFN resulted in decreased viability and migration of SGC-7901 cells, accompanied by enhanced G0/G1 phase arrest and alterations in protein expression related to cellular growth pathways [100]. In addition, SFN augmented the anti-cancer efficacy of sunitinib in renal cell carcinoma (RCC) by diminishing cell growth and proliferation. The combination treatment induced G2/M phase arrest by suppressing cyclin A, cyclin B, CDK1, p-CDK1, CDK2, and p-CDK2 in RCC cell lines [123].

Additionally, the combined treatment of SFN and cisplatin in H-28 malignant mesothelioma cells induces synergistic cell growth inhibition and promotion of apoptosis through ROS generation and modulation of ΔΨm, p53, caspase-3, and cell cycle proteins, suggesting a potential efficacy against mesothelioma (malignancy of tissue lining) [106].

Moreover, investigations into multiple myeloma (MM) have confirmed that a therapeutic approach combining SFN with arsenic trioxide (ATO) synergistically enhances the anti-neoplastic efficacy of ATO against MM by inducing PARP cleavage and increasing caspase-3 and -4 expression [102]. Moreover, the dual treatment disrupted protein homeostasis through ROS generation and elevated ER stress, leading to apoptosis [102]. Further, the concurrent treatment of SFN and TRAIL in Saos2 and MG63 osteosarcoma cells effectively overcame TRAIL resistance by significantly enhancing apoptosis through the induction of Bid cleavage, activation of caspases, and upregulation of DR5 levels [65]. In the aforementioned studies, SFN demonstrated synergistic actions with various chemotherapeutic agents across a range of cancers, highlighting its potential as a chemosensitizer and adjuvant in cancer therapy.

## 4. Conclusions and Future Directions

Current clinical research is extensively evaluating the prophylactic potential of natural compounds against carcinogenesis. Contemporary chemotherapeutic agents, while efficacious, are frequently associated with adverse side effects [144]. The incorporation of natural compounds in cancer preventive strategies may mitigate such adverse reactions [3]. SFN is emerging as a promising adjunctive therapeutic in oncology given its observed inhibitory effects on cancer cell growth and its role as a chemosensitizer.

Various studies on SFN demonstrate a promising potential as a chemosensitizer and as synergistic agent with other agents for the treatment and management of multiple cancers such as bladder, breast, colorectal, lung, pancreatic, prostate, and skin cancers. It has been found to enhance the efficacy of chemotherapy by sensitizing cancer cells to drugs like cisplatin, paclitaxel, and DTX, making them more responsive to treatment. SFN in combination with chemotherapeutic agents was found to regulate MAPK, Akt/mTOR, NF-κB, Wnt/β-catenin, and STAT3 signaling cascades, thereby inducing the reversal of drug resistance in cancer cells. Moreover, SFN combination with anti-cancer agents modulated various tumor suppressors, cyclins, MMPs, cadherins, anti-oxidant enzymes, stem cell markers, apoptotic regulators, etc., which lead to enhanced therapeutic outcomes of chemotherapeutics in cancer models both in vitro and in vivo. Additionally, combination studies involving the co-treatment of SFN with chemotherapeutic agents and other phytochemicals, peptides, suggest its efficacy in synergistically modulating various cancer hallmarks like survival, proliferation, invasion and migration by modulating various pathways. From the aforementioned studies on various cancers, it was also noted that SFN exhibits the ability to enhance the cytotoxicity of chemotherapy on cancer cells while also possibly minimizing the damage to normal cells and healthy tissues. Thus, the chemosensitizing efficacies of sulforaphane make it a potential candidate for cancer therapeutic research, as it may contribute to improved therapeutic outcomes by overcoming chemoresistance and enhancing the overall efficacy of chemotherapy and other combination agents.

However, while SFN exhibits anti-carcinogenic and synergistic properties, certain studies indicate that it may suppress T cell-mediated immune responses, potentially compromising the efficacy of immunotherapeutic interventions. This suggests the need for comprehensive investigations into SFN immunomodulatory effects [145]. The industrial production and commercial distribution of SFN are constrained by its instability, as it is vulnerable to oxygen, heat, and alkaline conditions. Nonetheless, gold nanoparticle-based formulations appear promising, suggesting the need for rigorous research to optimize SFN formulations [61]. In addition, SFN mediated epigenetic modulation of gene expression warrants deeper exploration, particularly concerning its chemosensitizing potential in cancer cells. A notable challenge associated with natural compounds like SFN is their suboptimal bioavailability [3]. The different strategies such as formulation and nanoencapsulation could be developed and explored to enhance its absorption and systemic delivery.

In conclusion, this comprehensive review delineates the significant potential of SFN in chemosensitization by enhancing the efficacy of chemotherapeutic agents and overcoming or reversing chemoresistance. The multitargeted mechanism through which SFN regulates key oncogenic pathways suggests its significance as adjunct in combination strategies employed to tackle cancers. While these findings encourage the potential of SFN, it is also imperative to conduct rigorous preclinical and clinical studies to validate and optimize for the dose, duration and safety profile of sulforaphane in cancer treatment procedures. However, it is also noteworthy that the inclusion of sulforaphane as a chemosensitizer holds a significant promise of advancing therapeutic management of cancers, ultimately contributing to improved outcomes in patients.

## Figures and Tables

**Table 1:** Mechanistic action of Sulforaphane in Chemosensitization and Combination Therapy.

Cancer	Sulforaphane in Combination with	In Vitro/In Vivo	Mechanism of Action/Anticancer Effect	Reference
ACC	5-FU *^A^	In vitro	↓NF-κB p65; ↓cell growth	[60]
Bladder Cancer	AZ *^A^	In vitro	↓Cell viability; ↓cell proliferation; ↓p-Akt; ↓colony formation; ↓Ki-67+ cells; ↓cyclin D1; ↓pHH3+ cells; ↓p-S6 ↓p-mTOR; ↓p-GSK-3-alpha/beta; ↑p21; ↑p27; ↑cleaved caspase-3; ↑cleaved PARP	[43]
	AZ *^A^	In vivo	↓Tumor weight; ↓CA9; ↓E-cadherin; ↓N-cadherin; ↓vimentin	[43]
	TRAIL *^C^	In vitro	↓Procaspase-3, -8, -9; ↓Nrf 2 (nuclear); ↑DR5; ↑cleaved PARP; ↑cleaved Bid; ↑ΔΨm loss; ***↑***apoptosis; ***↑***ROS	[70]
	Everolimus (long term) ^#A^	In vitro	↓Clone count; ↓CDK2; ↓p-CDK1; ↓cyclin B; ↓Raptor; ↓p-Rictor; ↓p-Akt; ↓cyclin A; ↓H3; ↑p19; ↓p27; ↑G0/G1 phase arrest; ↑CDK1; ↑Akt; ↑aH3; ↑aH4	[71]
Bone Cancer	TRAIL *^C^	In vitro	↑Apoptosis; ↑cleaved Bid; ↑cleaved caspase -3, -8, -9, -10; ↑DR5; ↑chromatin condensation	[65]
Brain Cancer	Resveratrol *^B^	In vitro	↓Colony formation; ↓p-Akt; ↓Akt; ↓cell migration; ↓PCNA; ↓cyclin D1; ↓cell viability; ↑Bax; ↑Cyt C; ↑cleaved caspase-3	[62]
	TMZ ^#A^	In vitro	↓Bcl-2; ↑Bax; ↑apoptosis; ↑caspase 3/7 activity	[44]
	TMZ ^#A^	In vivo	↓Tumor volume; ↓tumor weight; ↓Bcl-2; ↑Bax	[44]
	TMZ ^#A^	In vitro	↓Cell invasion; ↓cell proliferation; ↓MGMT; ↓NF-kB; ↑apoptosis; ↑caspase 3/7 activity	[64]
	TMZ ^#A^	In vivo	↓Tumor volume; ↓p65; ↓MGMT; ↓Ki-67; ↓MMP-2, -9; ↑caspase 3	[64]
	R8-PNAa15b *^C^	In vitro	↓Cell proliferation; ↓miR-15b-5p; ↑apoptosis	[72]
Breast Cancer	ClF *^A^	In vitro	↓Cell growth; ***↑***PTEN hypomethylation; ↑apoptosis; ↑p21; ↑RARβ-2	[73]
	Lapatinib ^#A^	In vitro	↓Cell viability; ↓p-HER2, ↓p-Akt, ↓p-S6; ↑caspase-3; ↑cleaved PARP; ↑apoptosis	[74]
	DTX-SFN-PLGA-b-HA ^#A^	In vitro	↓Cell viability; ↓β-catenin; ↓Cyclin D1	[75]
	DTX-SFN-PLGA-b-HA ^#A^	In vivo	↓Tumor volume, ↓tumor weight; ↓mammospheres; ↓β-catenin; ↓cyclin D1	[75]
	4-hydroxytamoxifen ^#A^	In vitro	↓Cell viability; ↓Bcl-2; ↓survivin; ↓colony formation; ↑ADRP; ↑cleaved PARP; ↑Bax; ↑LC3-II	[76]
	Paclitaxel ^#A^	In vitro	↓Cell viability; ↓NF-κB; ↓p-Akt; ↓ΔΨm; ↓IκBα degradation; ↓p-IKK; ↑apoptosis; ↑cleaved caspase-3, -8, -9; ↑Cyt C	[50]
	Paclitaxel/Docetaxel ^#A^	In vitro	↓IL-6, -8; ↓cyclin D1; ↓cell viability; ↓ALDH+ cells; ↓CD44+/CD24-/EpCAM+ cell; ↓primary and secondary mammospheres	[42]
	Paclitaxel/Docetaxel ^#A^	In vivo	↓Tumor volume; ↓secondary tumor formation; ↓tumor initiation ability	[42]
	ClF *^A^	In vitro	↓Cell viability; ↑CDKN2A mRNA	[77]
	DOX ^#A^	In vitro	↓DNMT; ↓HDAC; ↓ERα; ↑caspase-3	[78]
	DOX ^#A^	In vivo	↓Tumor volume	[78]
	WA *^B^	In vitro	↓Cyclin D1; ↓CDK4; ↓p-RB; ↑E2F; ↑p21; ↑G1 phase arrest; ↓HDAC2, 3; ↑global methylation	[52]
	GEN *^B^	In vitro	↓Cell viability; ↓KLF4; ↓cell density; ↓cell proliferation; ↓HDAC2; HDAC3; ↓HMTs; ↓hTERT; ↑G1 & G2/M phase arrest	[46]
	GEN *^B^	In vivo	↓Tumor volume; ↓tumor incidence	[46]
	DOX ^#A^	In vitro	↓Cell growth	[79]
	DOX ^#A^	In vivo	↓Tumor volume	[79]
	NaB *^B^	In vitro	↓Cell growth; ↓DNMT3A; ↓DNMT3B), ↓HDAC1; ↓HDAC6; ↓HDAC11, ↓EZH2; ↓SUV39H1; ↓GCN5; ↓PCAF; ↓P300; ↓CBP; ↑apoptosis	[80]
	(GEN + NaB) *^B^	In vitro	↓Cell viability; ↓DNMT3A, ↓DNMT3B; ↓HDAC 1, 6, 11; ↓EZH2; ↓SUV39H1; ↓GCN5; ↓PCAF; ↓P300; ↓CBP; ↓H3K9 me; ↓H3K27me; ↑HAT activity; ↑G2/M phase arrest; ↑apoptosis	[80]
	DOX ^#A^	In vitro	↓PGE2; ↓Cox-2; ↓MDSCs accumulation; ↓CD11b+Gr-1+ MDSCs; ↑Nrf2; ↑HO-1; ↑GCLC; ↑CD8+ IFN-γ +T cells; ↑CD8+ granzyme+ T cells	[81]
	DOX ^#A^	In vivo	↓Cox-2/PGE2; ↓tumor volume; ↓MDSCs accumulation	[81]
	DOX-lip *^A^	In vitro	↓Cell viability; ↓ROS; ↑DNA damage; ↑Nrf2	[82]
	DOX-lip ^#A^	In vivo	↓Tumor growth; ↓mitotic index; ↓inflammatory cell infiltration; ↓leukocyte; ↓CK; ↓CK-MB isoenzyme; ↓metastatic foci in lungs; ↑cytotoxicity; ↑granulocyte infiltration; ↑lymphocyte; ↑monocyte; ↑hemoglobin; ↑hematocrit; ↑RBC	[82]
	SFN-CDDP-NPs ^#A^	In vitro	↓GSH; ↓cell viability; ↓Bcl-2; ↓PARP; ↑γ-H2AX; ↑p53; ↑cleaved PARP; ↑apoptosis	[83]
	SFN-CDDP-NPs ^#A^	In vivo	↓GSH; ↓Bcl-2; ↓tumor growth; ↑p53; ↑cleaved PARP; ↑γ-H2AX; ↑apoptosis; ↑AST; ↑ALT	[83]
	Nano-MTFN *^A^	In vitro	↓WNT1; ↓β-catenin; ↓CD44; ↓cell survival; ↓Bcl-2; ↓Src; *↑*apoptosis; ↑Bax	[84]
	DOX ^#A^	In vitro	↓Cell viability	[85]
	Cisplatin ^#A^	In vitro	↓Cell proliferation; ↓cell migration; ↓cell invasion; ↓chemotaxis; ↓N-cadherin; ↓vimentin; ↓β-catenin ↓Slug; ↓TCF8/ZEB1; ↓Snail; ↓MMP-2, -9; ↓SIRT-1, -2, -3, -5, -7; ↓colony formation; ↓mammospheres; ↑claudin-1; ↑ZO-1; ↑S phase arrest; ↑E-cadherin; ↑apoptosis	[86]
	AT/DOX ^#A^	In vitro	↓Cell viability; ↓Bcl-2; ↑γ-H2AX; ↑Cyt-C; ↑cleaved PARP; ↑drug concentration; ↑cytotoxicity	[87]
	AT/DOX ^#A^	In vivo	↓Tumor volume; ↓Ki-67; ↑drug penetration; ↑apoptosis	[87]
Bronchial Carcinoma	AZ ^#A^	In vitro	↓Cell viability	[88]
	AZ ^#A^	In vivo	↓Tumor volume; ↓cell proliferation; ↓Oct-4; ↓Sox2; ↓nanog; ↓tumor weight; ↓tumor cells	[88]
Cholangiocarcinoma	Cisplatin ^#A^	In vitro	↓Cell viability; ↑cleaved caspase-3, ↑cleaved PARP; ↓Bcl-2; ↓XIAP	[89]
	GEM ^#A^	In vitro	↓Cell viability; ↓p-Cdc25C; ↓Bcl-2; ↓cell invasion; ↓cell migration; ↓CDH2; ↓vimentin; ↓MMP2, 9; ↓VEGFA; ↓VEGFR2; ↓HIF-1A; ↓NOS3; ↑G2/M phase arrest; ↑cleaved caspase-3; ↑p21; ↑p-Chk2; ↑Bax; ↑p21; ↑CDKN1A; ↑CDH1; ↑KRT19	[90]
	GEM ^#A^	In vivo	↓Tumor growth; ↓Ki-67+ cells; ↓p-Cdc25C; ↓VEGFA; ↓VEGFR2; ↓CDH2; ↓ vimentin; ↓MMP2, 9; ↓CD34^+^; ↑p21; ↑p-Chk2; ↑CDH1; ↑KRT19; ↑apoptosis	[90]
Cervical Cancer	Eugenol *^B^	In vitro	↓Cell viability; ↓Bcl-2; ↓Cox-2; ↓IL-β; ↑caspase-3	[91]
	(Eugenol ^B^ + GEM ^A^) *	In vitro	↓Cell viability; ↑caspase-3	[91]
Colorectal Cancer	Apigenin *^B^	In vitro	↑UGT1A1	[92]
	DIM *^B^	In vitro	↓Cell proliferation; ↑G2/M arrest; ↑cleaved PARP	[54]
	EGCG *^B^	In vitro	↓Cell viability; ↓cellular senescence; ↓cyclin D1; ↑AP-1	[93]
	OX ^#A^	In vitro	↓Cell proliferation; ↓ATP; ↓procaspase-8; ↑cleaved caspase-3, -8; ↑cleaved PARP; ↑necrosis; ↑DNA fragmentation; ↑TRAIL; ↑mitochondrial membrane depolarization	[57]
	*Lactobacillus*-treated PMBC *^C^	In vitro	↓XIAP; ↓cIAP-1, -2; ↑apoptosis; ↑mitochondrial membrane depolarization; ↑TNF-α; ↑TNF-R1; ↑Bax	[47]
	SAL *^A^	In vitro	↓Cell viability; ↓cell proliferation; ↓p-Akt; ↓Bcl-2; ↓PI3K; ↓cell migration; ↓cell invasion; ↑p53; ↑Bax; ↑cleaved PARP; ↑apoptosis	[49]
	SAL *^A^	In vivo	↓Tumor growth, volume, weight	[49]
	PNAs *^C^	In vitro	↓Cell growth; ↑apoptosis; ↑caspase-3; ↑Bak1; ↑p53	[94]
	(Lycopene+ Quercetin+ Curcumin) *^B^	In vitro	↓Cell proliferation; ↓DNA synthesis	[95]
	(Lycopene ^B^ + Quercetin ^B^ + Curcumin ^B^ + 5-FU ^A^) ^#^	In vitro	↓Cell proliferation	[95]
	(Lycopene ^B^ + Quercetin ^B^ + Curcumin ^B^ + Cisplatin ^A^) ^#^	In vitro	↓Cell proliferation	[95]
	CB-5083 ^#A^	In vitro	↓Cell proliferation; ↓cell colonies	[96]
	FOLFOX ^#A^	In vitro	↓ALDH1; ↓cell viability; ↓spheroid formation; ↑apoptosis	[97]
	Vitamin D ^*C^	In vivo	↓Tumor size; ↓HDAC6; ↑LC3II	[98]
Epidermal Squamous Cell Carcinoma	Cisplatin ^#A^	In vitro	↓Spheroid formation; ↓cell invasion; ↓wound closure; ↓cell number; ↓p21^cip1^; ↓PARP; ↑cleaved caspase-3, -9; ↑cleaved PARP; ↑apoptosis	[99]
	Cisplatin ^#A^	In vivo	↓p21^cip1^; ↓tumor volume; ↑cleaved caspase-3; ↑cleaved PARP	[99]
Gastric Cancer	Lapatinib ^#A^	In vitro	↓Cell viability; ↓cell migration; ↓HER2; ↓p-HER2; ↓Akt; ↓p-Akt; ↓ERK; ↓p-ERK; ↑apoptosis; ↑G0/G1 phase arrest	[100]
Head and Neck Cancer	Cisplatin ^#A^	In vitro	↓Spheroid formation; ↓BMI1; ↓cell viability; ↓OCT4; ↓Sox2; ↓Bcl-2; ↓ALDH1A1; ↓Notch1; ↓SMO; ↓GLI1; ↑caspase-3; ↑apoptosis	[101]
	Cisplatin ^#A^	In vivo	↓Tumor volume	[101]
	5-FU ^#A^	In vitro	↓Spheroid formation; ↓BMI1; ↓Sox2; ↓Bcl-2; ↓ALDH1A1; ↓Notch1; ↓SMO; ↓GLI1; ↓cell viability; ↑caspase-3; ↑apoptosis	[101]
Multiple Myeloma	ATO ^#A^	In vitro	↓GSH; ↓secreted GLUC; ↓cell proliferation; ↓ARP1; ↓KMS11; ↑cleaved PARP; ↑cleaved caspase -3, -4; ↑ROS; ↑HSP90; ↑p-PERK; ↑p-eIF2; ↑CHOP; ↑spliced Xbp-1; ↑ER stress	[102]
Liver Cancer	TRAIL *^C^	In vitro	↓Cell viability; ↓XIAP; ↑ROS; ↑Bid; ↑cleaved PARP; ↑DR5; ↑apoptosis; ↑DNA fragmentation; ↑cleaved caspase-2, -3, -7, -8, -9	[103]
Lung Cancer	Cisplatin ^#A^	In vitro	↓Cell viability; ↑apoptosis; ↓c-Myc ↓spheroid formation	[104]
	Cisplatin ^#A^	In vivo	↓Tumor weight; ↓tumor volume; ↓c-Myc	[104]
	DOX *^A^	In vitro	↓c-Myc	[104]
	Gefitinib ^#A^	In vitro	↓Cell proliferation; ↓GLI1; ↓SMO; ↓SHH; ↓CD44; ↓CD133	[105]
	Gefitinib ^#A^	In vitro	↓PI3/Akt; ↓cell proliferation; ↓cell survival; ↓vimentin; ↓EGFR; ↓p-EGFR; ↓p-Akt; ↓p-ERK; ↓N-cadherin; ↑E-cadherin; ↑claudin-1; ↑G1 phase arrest; ↑apoptosis	[45]
	AITC *^B^	In vitro	↓Survivin; ↓cyclin B1; ↓Cox-2; ↓cell viability; ↓MMP-9; ↓p-STAT3; ↑migration; ↑p53; ↑cleaved caspase-3; ↑cleaved PARP; ↑G2/M phase arrest; ↑p21; ↑ROS; ↑apoptosis	[51]
Mesothelioma	Cisplatin ^#A^	In vitro	↓Cell viability; ↓Bcl-2; ↓p-Akt; ↓p-mTOR; ↓cyclin D1; ↓GSH/GssG; ↑p53; ↑cleaved caspase-3; ↑cleaved PARP; ↑apoptosis; ↑Bax; ↑p-Cdc2^tyr15^; ↑ROS; ↑ΔΨm loss; ↑autophagy; ↑LC3B-II; ↑sub G1 arrest; ↑cyclin B1	[106]
Neuroblastoma	3-MA *^A^	In vitro	↓Cell viability; ↓LDH; ↓Bcl-2; ↑cell death; ↑LC3-I; ↑LC3-II; ↑ΔΨm loss	[107]
Ovarian Cancer	(EGCG ^B^ + Cisplatin ^A^) ^#^	In vitro	↓Cell viability; ↓cell proliferation; ↑G2/M phase arrest; ↑p21; ↑drug efficacy; ↑apoptosis	[108]
	EGCG *^B^	In vitro	↓Cell viability; ↓hTERT; ↓DNMT1; ↓telomerase activity; ↑G2/M & S phase arrest; ↑apoptosis; ↑p-H2AX	[109]
	(EGCG ^B^+ Paclitaxel ^A^) ^#^	In vitro	↓Cell viability; ↓cell proliferation; ↓colony formation; ↓hTERT; ↓DNMT1; ↓telomerase activity; ↑G2/M & S phase arrest; ↑apoptosis; ↑cleaved PARP; ↑p-H2AX	[109]
	Cisplatin ^#A^	In vitro	↓GSH; ↑cytotoxicity; ↑apoptosis; ↑GCLC; ↑Nrf-2	[110]
	Cisplatin *^A^	In vitro	↓Cell proliferation; ↓Bcl-2; ↓cyclin D1; ↓c-Myc; ↓colony formation; ↓cells in G2/M/S phase; ↑p53; ↑caspase 3	[111]
	Cisplatin ^#A^	In vitro	↓ERCC1; ↓ATP7A; ↑miR-30a-3p; ↑DNA damage; ↑cytotoxicity; ↑drug concentration	[112]
	Cisplatin ^#A^	In vivo	↓Tumor volume; ↓ERCC1; ↓ATP7A; ↑miR-30a-3p	[112]
	Cisplatin ^#A^	In vitro	↓c-Myb	[113]
Pancreatic Cancer	SO ^#A^	In vitro	↓NF-κB; ↓cIAP; ↓XIAP; ↓cFLIP; ↓colony & spheroid formation; ↓survival fraction; ↑cell death; ↑caspase- 3/7; ↑caspase-8, -9; ↑ALDH^+^ cells	[114]
	SO ^#A^	In vivo	↓Tumor growth; ↓Zeb-1; ↓Twist2; ↓vimentin; *↓*HIF-1α	[114]
	17-AAG ^#A^	In vitro	↓Cell viability; ↓Akt; ↓mut p53; ↓Raf-1; ↓CDK4; ↑caspase-3	[115]
	17-AAG ^#A^	In vivo	↓Tumor growth; ↓tumor weight; ↓tumor volume	[115]
	(ASP ^A^ +CUR SLN ^B^) *	In vitro	↓Cell viability; ↑apoptosis	[116]
	(ASP ^A^ +CUR ^B^) *	In vitro	↓Cell viability; ↓p-IκBα; ↓p-Akt; ↓survival fraction; ↓NF-κB activity; ↑apoptosis; ↑cleaved caspase-3; ↑p-ERK1/2; ↑p-c-Jun; ↑p-p53; ↑p-p38 MAPK; ↑cleaved PARP	[48]
	LOR SMEDDS *^A^	In vitro	↓Cell inhibition	[117]
	GTC *^A^	In vitro	↓Colony formation; ↓K-ras; ↓spheroid formation; ↓ALDH1^+^ cells; ↓cell viability; ↓cell migration; ↓MMP-2, -9; ↓survival fraction; ↑miR-let7-a; ↑apoptosis	[118]
	LOR SMEDDS *^A^	In vitro	↓Colony formation	[119]
	(ASP ^A^ + CUR ^B^) *	In vivo	↓Tumor incidence; ↓tumor progression	[120]
Prostate Cancer	TRAIL *^C^	In vitro	↓NF-κB; ↓survival fraction; ↓spheroid formation; ↓Nanog; ↓CXCR4; ↓jagged1; ↓CD44; ↓CD133; ↓CXCR4; ↓EpCAM; ↓c-Met; ↓Ki-67; ↓Notch 1; ↓Sox2; ↓ALDH1 activity; ↓cell differentiation; ↑cleaved caspase-3	[121]
	TRAIL *^C^	In vivo	↓Tumor growth; ↓CD44; ↓CD133; ↓Nanog; ↓CXCR4; ↓EpCAM; ↓c-Met; ↓ALDH1 activity; ↓Ki-67; ↑caspase-3	[121]
	Paclitaxel *^A^	In vitro	↑Apoptosis	[122]
Renal Cell Carcinoma	Sunitinib *^A^	In vitro	↓Cell growth; ↓CDK1; ↓p-CDK1; ↓CDK2; ↓p-CDK2; ↓clonogenic growth & proliferation; ↓cyclin A; ↓cyclin B; ↑drug efficacy; ↑G2/M phase arrest	[123]
Skin Cancer	Quercetin *^B^	In vitro	↓Cell viability; ↓cell migration	[124]
	Quercetin *^B^	In vivo	↓Tumor volume; ↓MMP9	[124]
	DAC *^A^	In vitro	↓Cell viability; ↓cell growth; ↑CCL5; ↑IL-33; ↑DUSP15; ↑CXCL10; ↑angiopoietin-2; ↑CD105; ↑VEGF; ↑CCN4	[125]
	Nano-CUR *^B^	In vitro	↓Cell viability	[126]
	FB *^A^	In vitro	↓Cell growth; ↓MMP-1, -2, -3, -9; ↓Cell migration; ↓IL-1β; ↓VEGF; ↓NLRP3; ↓ASC; ↓cleaved caspase-1	[127]

* Combination; ^#^ chemosensitization; ^A^ FDA approved/patented drugs; ^B^ Phytochemicals/compounds; ^C^ Biological molecules/peptides/microbes/others. 5-FU—5-fluorouracil; 17-AAG—17-allylamino 17-demethoxygeldanamycin; ACC—adenoid cystic carcinoma; aH—acetylation of histone; ADRP—adipocyte differentiation-related protein; AITC—allyl isothiocyanate; ALDH1—aldehyde dehydrogenase 1; AR—androgen receptor; ASC—apoptosis-associated speck-like protein containing a caspase recruitment domain A; ASP—aspirin; ATO—arsenic trioxide; AZ—acetazolamide; Bcl-2—B-cell lymphoma 2; BITC—benzyl isothiocyanate; ClF—clofarabine; CA-9—carbonic anhydrase-9; CB-5083—p97 inhibitor drug; CUR—curcumin; DIM-3,3′-diindolylmethane; DNMT—DNA methyltransferase; DNMT-2-chloro-2′-fluoro-2′-deoxyarabinosyladenineDNA; DOX-lip—doxorubicin-liposomal; DOX—doxorubicin; DR5—death receptor 5; DTX—docetaxel; DTX-SFN-PLGA-b-HA)-NPs -docetaxel (DTX)- and sulforaphane (SFN)-loaded poly(D, L-lactide-coglycolide)/hyaluronic acid-based nanoparticle; EGFR—epidermal growth factor receptor; EGCG—epigallocatechin gallate; ERα—estrogen receptor alpha; ERK—extracellular signal-regulated kinase; EZH2—enhancer of zeste homolog 2; FB—fernblock^®^ XP; FU—fluorouracil; GCLC—glutamate-cysteine ligase; GEN—genistein; GSH—glutathione; GTC—green tea catechins; HDAC—histone deacetylase; HMT—histone methyl transferase; Hsp 90—heat shock protein 90; IM—imatinib; 3-MA—3-methyladenine; KLF4-krüppel-like factor 4; LOR—loratadine; LOR SMEDDS-SFN—loratadine self-microemulsifying drug delivery system–sulforaphane; MDSC—myeloid-derived suppressor cell; MGMT-O6—methylguanine-DNA methyltransferase; ΔΨm—mitochondrial membrane potential; mutp53—mutant p53; NLRP3-nucleotide-binding domain, leucine-rich-containing family, pyrin domain-containing-3; NaB—sodium butyrate; NP—nanoparticle; Nano-MTFN—nano-Metformin; Nrf2—NF-E2-related factor 2; OCT4—ocatamer-binding transcription factor 4; OXP—oxaliplatin; PARP—poly(ADP-ribose) polymerase, PGA-CDDP—polyethylene glycol *cis*-dichlorodiammineplatinum(II); pHH3—phospho histone H3; PCNA—proliferating cell nuclear antigen; PDT—photodynamic therapy; p-GSK-3: glycogen synthase kinase 3; PMBC—peripheral blood mononuclear cells; PNAs—peptide-nucleic acids; p-RB—phosphorylated RB; PTEN—phosphatase and tensin homolog; RB—retinoblastoma protein; RBC—red blood cell; SAL—salinomycin; Ser9-phospho-GSK3β; Ser37—dephosphorylated β-catenin; SIRTs—sirtuins; SLN: solid lipid nanoparticle; Se-NPs—selenium nanoparticles; SHH-sonic Hedgehog SO-sorafenib; SUV39H1—suppressor of variegation 3–9 homolog 1; TMZ—temozolomide; TS—thymidylate synthetase; UGT—UDP-glucuronosyltransferases; WA—withaferin A.

## Abbreviations

Bak1	Bcl-2 homologous antagonist/killer
Bax	Bcl-2-associated X protein
Bcl-2	B-cell lymphoma 2
CDK	Cyclin dependent kinase
CHOP	C/EBP Homologous Protein
cPARP	Cleaved Poly(ADP-ribose) polymerase
EMT	Epithelial-mesenchymal transition (EMT)
GLS	Glucosinolates
IL	Interleukin
LC3-II	Microtubule-associated protein 1A/1B-light chain 3-II
NF-κB	Nuclear factor kappa B
Nrf2	NF-E2-related factor 2
ROS	Reactive oxygen species
SFN	Sulforaphane
TNF-α	Tumor necrosis factor-alpha
VEGF	Vascular endothelial growth factor
